# Piezoelectric Biosensors for Organophosphate and Carbamate Pesticides: A Review

**DOI:** 10.3390/bios4030301

**Published:** 2014-09-09

**Authors:** Giovanna Marrazza

**Affiliations:** Department of Chemistry “Ugo Schiff”, University of Florence, Via della Lastruccia 3, 50019 Sesto Fiorentino, Florence, Italy; E-Mail: giovanna.marrazza@unifi.it; Tel.: +39-055-4573320; Fax: +39-055-4574913

**Keywords:** pesticide, organophosphate, carbamate, biosensor, piezoelectric

## Abstract

Due to the great amount of pesticides currently being used, there is an increased interest for developing biosensors for their detection. Among all the physical transducers, piezoelectric systems have emerged as the most attractive due to their simplicity, low instrumentation costs, possibility for real-time and label-free detection and generally high sensitivity. This paper presents an overview of biosensors based on the quartz crystal microbalance, which have been reported in the literature for organophosphate and carbamate pesticide analysis.

## 1. Introduction

Pesticides play an important role in the high productivity achieved in agriculture through the control of plants or animals life that are considered pests. The Food and Agriculture Organization (FAO) has defined pesticide as: any substance or mixture of substances intended for preventing, destroying, or controlling any pest, including vectors of human or animal disease, unwanted species of plants or animals, causing harm during or otherwise interfering with the production, processing, storage, transport, or marketing of food, agricultural commodities, wood and wood products or animal feedstuffs, or substances that may be administered to animals for the control of insects, arachnids, or other pests in or on their bodies [1].

Although pesticides have benefits, some also have drawbacks, such as potential toxicity to humans and other desired species. Exposure of the general population to pesticide most commonly occurs through consumption of treated food sources. Persistent chemicals can be magnified through the food chain and have been detected in products ranging from meat and fish, to vegetable oils, various fruits and vegetables.

The levels of pesticide residues in foods are often stipulated by regulatory bodies in many countries. Some countries use the International Maximum Residue Limits-Codex Alimentarius to define the residue limits; this was established by Food and Agriculture Organization of the United Nations and World Health Organization (WHO) in 1963 to develop international food standards, guidelines codes of practices, and recommendation for food safety [2].

At present, the identification and quantification of pesticides are generally based on chromatographic methods, such as gas chromatography (GC) or high-performance liquid chromatography (HPLC) coupled with mass spectroscopy (MS) requiring a previous appropriate sample preparation [3,4,5].

Although accurate and reliable, above traditional techniques involve time-consuming steps such as field sample collection, solid-phase extraction in laboratory, analyzing the sample, and comparing the obtained spectral peaks with references to determine the pesticide residues. Thus, researchers have been investigating alternative methods of detection and screening that are cheaper and more user-friendly.

In recent years, there has been an increasing interest in biosensor technology for fast pesticide detection using easy and rapid procedures. The biosensors have the potential to complement or replace the classical analytical methods by simplifying or eliminating sample preparation and making field-testing easier and faster with significant decrease in cost per analysis. A wide variety of them reported for different food and environmental applications exist and excellent reviews are described in the literature [6,7,8,9,10].

As many pesticides are designed to inhibit various enzymes within insects and other pests, utilizing these enzymes for detection purposes seemed a logical route. In this manner, enzymes such as acetylcholinesterase, butyrylcholinesterase and others were investigated for their ability to detect pesticides in environment [11,12,13,14].

Among the biosensors, piezoelectric sensors have attracted research interest and served as alternatives to the conventional immunoassay tools for detecting pesticides. They offer advantages such as real-time output, high sensitivity, simplicity of use, and cost-effectiveness. Moreover, they can be designed without the need of expensive or hazardous labels.

As families, organophosphate and carbamate compounds represent a large number of pesticides and insecticides. Because of their low persistence and high effectiveness, they are widely employed in agriculture. Although they have low environmental persistence and high effectiveness, some exhibit high acute toxicity in humans. Based on various signal transduction mechanisms, the current existing pesticide biosensor devices primarily use enzyme-linked immunosorbent assay (ELISA), surface plasmon resonance (SPR) [15], electrochemical enzyme sensor [11]. These devices allow for sensitive detection with a limit-of-detection (LOD) range of 10^−7^–10^−8^ M. Focusing on the recent activity of worldwide researchers, without pretending to being exhaustive, the aim of the present review is to give the readers a critical overview of piezoelectric biosensors based on quartz crystal microbalance for organophosphorus and carbamate pesticides analysis. In the Section 2, organophosphate and carbamate proprieties are discussed. In the Section 3, the principle of Quartz Crystal Microbalance is presented. The Section 4 is dedicated to immobilization procedures of bioreceptors onto sensor surface. In the Section 5, various biosensors measuring organophosphate and carbamate pesticides are reviewed. Table 1 summarizes the specific pesticides tested using various types of analytical methods, integrated with piezoelectric quartz transducers, and the limits of detection in each case. Finally, future considerations and opportunities for advancing the use of biosensors to monitor pesticides are discussed.

**Table 1:** Biosensors based on quartz-crystal microbalance for pesticides detection.

Pesticide	Detection Limit	Reference
Carbaryl Paraoxon	1 × 10^−7^ M 5 × 10^−8^ M	[16]
Carbaryl Dichlorvos	1 mg/L 1 mg/L	[17]
Carbofuran EPN	1.30 × 10^−9^ M 1.55 × 10^−8^ M	[18]
Diisopropylfluorophosphate	1 × 10^−10^ M	[19]
Paraoxon Diisopropylfluorophosphate Chlorpyriphos Chlorfenvinphos	1 × 10^−10^ M	[20]
Carbofuran	4.5 × 10^−6^ M	[21]
Metolcarb	0.019 mg/L	[22]
Parathion	4 μg/L	[23]
Carbaryl TCP	11 μg/L 7 μg/L	[24]
Carbaryl	2 × 10^−10^ M	[25]
Glyphosate Chloropyrifos Methyl Diazinon	250 μg/L	[26]
Pirimicarb	5 × 10^−7^ M	[27]
Endosulfan	5.59 μg/L	[28]
Paraoxon	6 × 10^−8^ M	[29]

## 2. Organophosphate and Carbamate Proprieties

**Figure 1:**
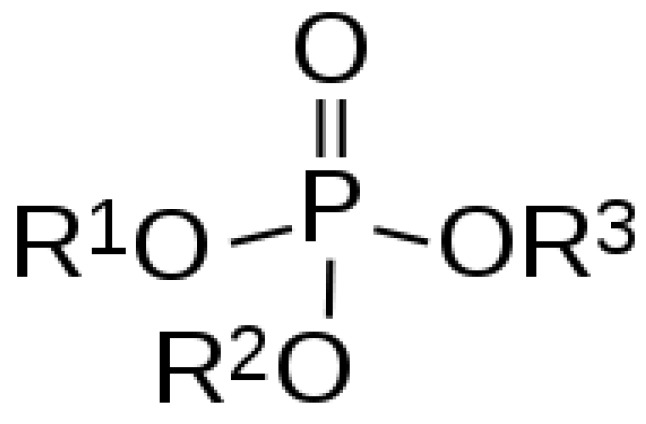
Chemical structure of Organophosphates.

Organophosphates (OPs) are usually esters, amides or thiol derivatives of phosphoric, phosphonic, or phosphinic acids, which have general structural formula (Figure 1) where R^1^ and R^2^ are alkyl-, alkoxy, alkylthio-, or amido-groups. X is the acyl residue (labile fluorine-, cyano-, substituted or branched aliphatic, aromatic, or heterocyclic groups).

They were developed during the early 19th century, but their effects on insects, which are similar to their effects on humans, were discovered in 1932. Some are very poisonous; they were used in World War II as nerve agents.

Commonly used organophosphate pesticides include parathion, malathion, methyl parathion, chlorpyrifos, diazinon, dichlorvos, phosmet, fenitrothion, tetrachlorvinphos and azinphos methyl.

**Figure 2:**
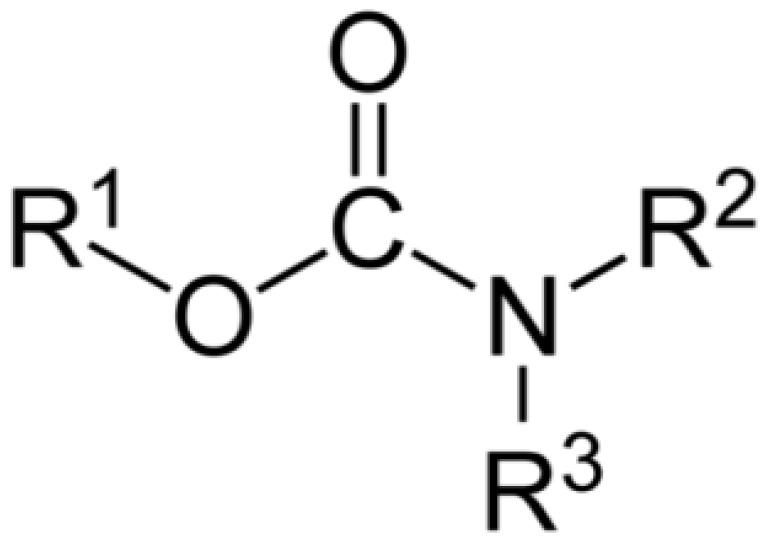
Chemical structure of Carbamates.

The so-called carbamate insecticides feature the carbamate ester functional group derived from carbamic acid (NH_2_COOH). Included in this group are aldicarb, carbofuran, carbaryl, ethienocarb, fenobucarb, oxamyl and methomyl. The general chemical structure is shown in Figure 2.

Organophosphate and carbamate compounds act by inhibition of acetylcholine esterase (AChE) activity, which is essential for the functioning of the central nervous system (CNS) of humans and insects. This causes the accumulation of the acetylcholine (ACh) neurotransmitter, which interferes with muscular responses and causes respiratory and myocardial malfunctions and even death. The sensitive, rapid and reliable determination of these compounds in environmental samples is therefore important for the protection of the environment and of human health [30,31,32,33]. The toxicity of these chemical compounds varies considerably, depending on the chemical structure of the pesticide. Many electrochemical biosensors for organophosphate and carbamate pesticide analysis are based on the principle of inhibition of cholinesterase [34,35,36,37,38,39].

## 3. Quartz Crystal Microbalance: Principle

**Figure 3:**
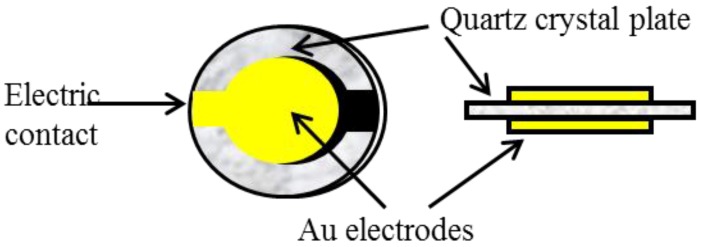
Schematic of a piezoelectric quartz crystal.

The quartz crystal microbalance (QCM) technique that uses a mass-sensitive detector based on an oscillating piezoelectric quartz crystal that resonates at a fundamental frequency by the application of an external alternating electric field. QCMs are piezoelectric devices fabricated of a thin plate of quartz, with gold electrodes affixed to each side of the plate (Figure 3).

Piezoelectric transducers are being used as chemical sensors since the discovery of the relationship between mass deposited/adsorbed on the crystal surface and the resonant frequency variations. This relationship is expressed by Sauerbrey’s equation:

ΔF = −KF^2^Δm/A^−1^ = −2.3 × 10^6^ F^2^Δm/A^−1^ (1)

where ΔF is the change of frequency (Hz) due to coating, k is the proportional constant depending upon density and shear modulus of quartz crystal (for AT-cut quartz, the density is 2.648 g/cm^3^ and shear modulus is 2.947 × 10^11^ dynes/cm^2^), F is the fundamental frequency (MHz) of the quartz crystal, Δm is mass (g) of coating deposited, and A is the coated area of the crystal (cm^2^).

This Sauerbrey equation, however, holds only for the case of rigid coated material. The frequency responses are also influenced by many factors, such as effective viscosity, conductivity, dielectric constant, electrode morphology, density and temperature of the liquid and ionic status of a crystal/electrode interface to a water/buffer. Thus, when used practically, operating conditions should be evaluated.

**Figure 4:**
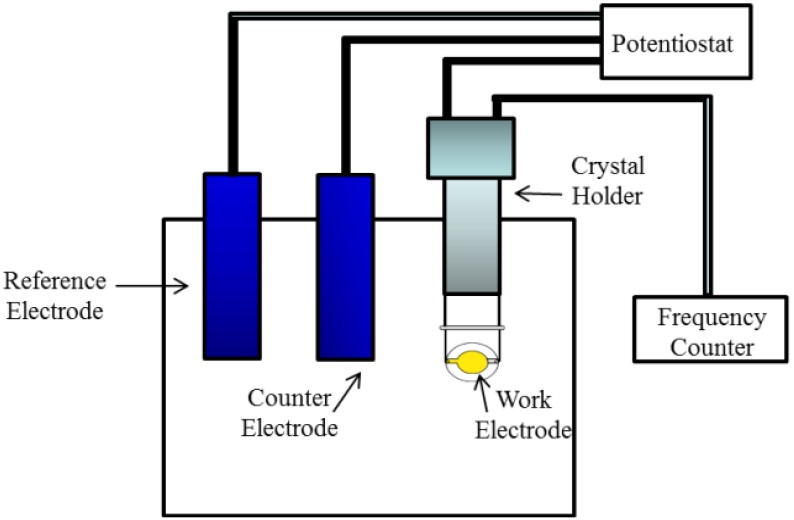
Schematic electrochemical quartz crystal microbalance (EQCM) apparatus.

In most electrochemical experiments, mass changes occur as material is deposited or lost from the “working” electrode. It is of interest to monitor those changes simultaneously with the electrochemical response. As a gravimetric probe, the QCM has been used in many types of electrochemical studies, including: underpotential deposition of metals, corrosion, oxide formation, dissolution studies, adsorption/desorption of surfactants and changes in conductive polymer films during redox processes. A schematic diagram of the apparatus for electrochemical quartz crystal microbalance (EQCM) experiments is given in Figure 4.

QCM crystal is fixed on a crystal holder with only one electrode exposed to the conductive solution. The crystal holder is placed in the cell with the working electrode and the counter electrode and connected to a control unit, which is in turn attached to a potentiostat. A PC is often interfaced to the potentiostat/frequency counter, and integrates the QCM and electrochemical data into a single software package.

**Figure 5:**
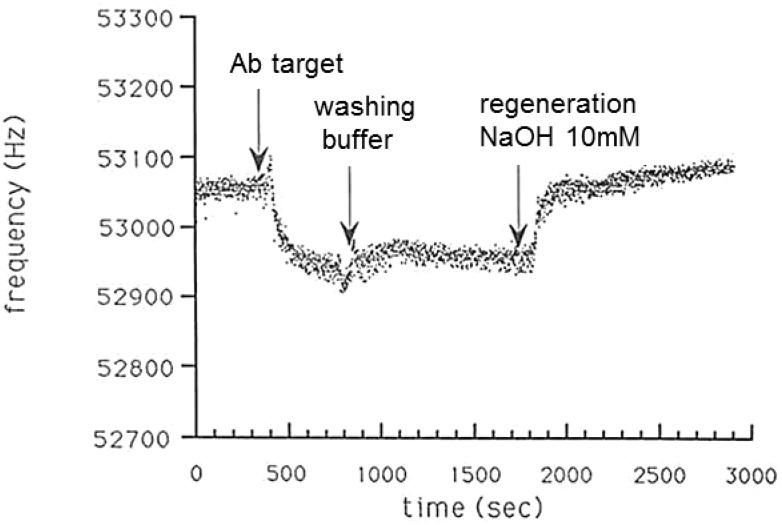
Scheme of the relation between action on sensor surface and resulting frequency *versus* time curve, showing an antibody–antigen reaction as an example.

Because of their simplicity, piezoelectric quartz crystal sensors have been a great importance as competitive tools for bioanalytical assays and for the characterization of biomolecular interactions. In these biosensors, the bioreceptor is immobilized on quartz crystal, which resonate on application of an external alternating electric field. The biospecific reaction between the two interactive molecules, one immobilized on the surface and the other free in solution or gas phase, can be followed in real time. Samples are usually applied to the immobilized ligand on the sensor surface by a continuous constant flow. A constant analyte concentration at any part of the flow through cell is thus provided, and diffusion effects can be neglected. Moreover, an optimized cell design helps to avoid mass transport effects. Viscosity effects, however, can lead to frequency shifts interfering with the specific signal. Therefore, the biosensor should be calibrated with a viscous solution to determine the time in which the viscosity change generates a signal. This time interval should be excluded from the sensorgram. A sample sensorgram is given in Figure 5. After a measurement, the sensor has to be regenerated to remove bound analyte from the sensor surface. For this regeneration step, elution buffers as used in affinity chromatography can be applied as long as they do not destroy the native structure of the ligand. It should be noted that the QCM method does not allow the measurement of true affinities. Because the ligand is immobilized, one degree of freedom is lost. Therefore, the measured affinities could be influenced by decreased mobility and sterical hindrance. Furthermore, avidity effects resulting from multivalent binding might affect the apparent affinity. On the other hand, partial denaturation of the immobilized ligand may decrease the apparent binding affinity. Nevertheless, as real-time measurements are performed, the sensorgrams are capable of deducing not only the equilibrium binding constants but also the affinity rate constants.

Piezoelectric transducers have been widely applied embraced for immunosensing applications. The advantages of using this transducer is real time monitoring, label free detection and simplicity of use. However, there are some drawbacks needing to be overcome such as lack of specificity and sensitivity as well as excessive interference. In addition, the piezoelectric biosensors have format and calibration problems. For this reason, that the piezoelectric sensors are not receiving much attention and are considered inferior compared to electrochemical and optical biosensors. However, the application range of the quartz crystal is in progress. New measuring techniques such as atomic force microscope (AFM) that use the quartz crystal as a transducer for chemical sensors and biosensors has been also developed [40]. New research results are expected to be continuously announced.

## 4. Immobilization Procedures of Bioreceptors

A great variety of methods for molecular bioreceptor immobilization on the crystal surface has been reported in the literature. In protein-sensing devices, the immobilized compound determines the specificity of the device, and the immobilization method frequently influences parameters such as lower detection limit, sensitivity, dynamic range, reusability or liability for unspecific binding. The immobilization strategies most generally employed are physical or chemical methods. The choice of the immobilization method is dependent on the chosen assay format and detection principle.

**Figure 6:**
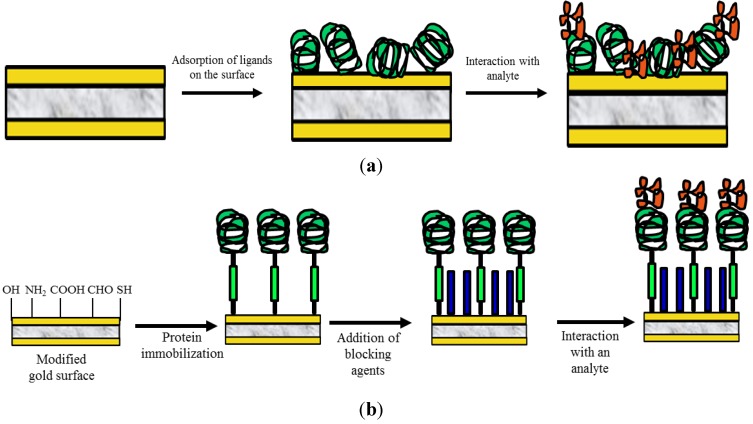
(**a**) Adsorption immobilization scheme. (**b**) General route for covalent immobilization of bioreceptors.

Physical adsorption on the solid surface is the most simple and fastest approach (no reagents or bioreceptor modifications are evolved). This method is based in weak interactions like Van der Waals, hydrogen bonding, hydrophobic or electrostatic interactions (Figure 6a). Van der Waals interactions are based in dipole-dipole attractions. Biomolecules can create positive or negative dipoles in originally non polar areas due to intramolecular interactions that disturb the electron clouds. When the biomolecule are immobilized, their dipoles align to maximize the interaction with the electric dipoles of the molecules in the surface. Hydrogen bonding occurs when a hydrogen atom covalently bound to an electronegative element is attracted by another electronegative element creating a relatively strong interaction.

The hydrophobic interactions are related to the presence of amino acids as phenylalanine and leucine that are non-polar and hence interact poorly with polar molecules like water. For this reason, most of the non-polar residues are directed toward the interior of the molecule whereas such polar groups as aspartic acid and lysine are on the surface exposed to the solvent. When the surface is functionalized with a hydrophobic layer, it is energetically more favorable for the non-polar residues to approach the surface creating a hydrophobic interaction.

Electrostatic interaction or physical adsorption is a simple process with the benefits of time saving and reduced complexity of ligand preparation. Its relative simplicity gives this approach certain advantages over the more complex covalent immobilization methods. However, the immobilization approaches result in a random orientation of the biomolecules since the orientation of the binding sites is not controlled. In addition, the biomolecules immobilization can be disturbed by pH or temperature changes. This results in a strong non-specific interaction between the sensor surface and bioreceptors which leads to decreased detection selectivity; confirming the validity of the method, the non-specific signals are difficult to be minimized.

The covalent attachment, affinity immobilization and self-assembling are, to date, the most successful approaches.

Bioreceptor is covalently linked through formation of a stable covalent bond between functional groups of protein and the transducer surface (Figure 6b).

The procedure can lead to ordered sets of end-point attached and properly oriented binding sites. Moreover, such chemistries also allow controlling the conformational freedom of the bioreceptors and the corresponding inter-chain space through the modulation of the surface coverage.

As the gold reacts with thiols, yielding a stable, semi-covalent bond, proteins can be immobilized by the thiol groups of their cysteine residues. Alternatively, the sensor surface can be activated by using a thiol-containing bifunctional linker. The linker layer serves as a functionalized structure for further modification of the surface, as well as creates a barrier to prevent proteins, DNA and other ligands from coming into contact with the metal. The linker, in fact, forms disulfide bonds to the gold surface and provides N-hydroxysuccinimide (NHS) groups that can react with the free aminogroups on the ligand. If streptavidin is immobilized using thiol-containing bifunctional linker, biotinylated ligands can be conveniently coated.

In comparison with the electrostatic interaction or physical adsorption immobilization techniques discussed above, covalent binding is normally more complex, sometimes requiring intensive synthesis work on the ligands. The procedure provides increased stability of the protein but decreases the activity of affinity reaction and is generally poorly reproducible.

The most used technique in piezoelectric sensor is based on Self-Assembled Monolayer (SAM) formation. The reported procedure for immobilizing ligands to gold surfaces is based on covalent bond formation between the sulphur containing ligands (*i.e*., thiol, disulfide and thiolester) and the metal atoms on the solid surfaces. The organothiol ligands form generally well ordered and crystalline monolayers on the metal surfaces. For example, when exposed to the gold surface, normally in an ethanolic solution, the sulphur-gold bond is immediately formed, but it takes several hours to reach the crystalline close parking of the layer. The chain length is very important for the monolayer quality. Organothiols with more than 10 methylene groups give better protection to the sulphur-gold bond and avoid oxidation, thus creating stable and reproducible monolayers.

In addition to these immobilization methods, new materials, such as nanoparticles and modified polymer have been employed.

Moreover, synthetic bioreceptors such as molecular imprinted polymer are being explored as tools affinity biosensor applications, as alternatives to traditionally used polyclonal or monoclonal antibodies.

A QCM analysis in a general protocol is broken down into the following steps: sensor cleaning, immobilization of the ligand on the sensor, sample preparation, system calibration, measurement procedure, optimization of regeneration buffer, and evaluation of sensorgram. Sensor cleaning and immobilization of ligand are performed before the sensor chip is mounted onto the device. Sensor cleaning should be performed directly before immobilization. The sensor chip with the immobilized ligand can be stored for several days at 4 °C in a wet chamber (storage conditions depend on the immobilized ligands).

## 5. Applications of Piezoelectric Biosensors in the Environment

The analytical technology based on sensors is an extremely wide field, which impacts on all the major industrial sectors, such as pharmaceutical, healthcare, food, agriculture, environment and water. The scope of this review is limited to piezoelectric biosensors developed over recent years specifically for determining analytes implicated in environmental and food analysis are reported.

They are divided in three categories in according to the molecular bioreceptor used for biosensor designing.

### 5.1. Detection of Pesticides Using Enzymatic Methods

The first biosensor based on cholinesterase (ChE) inhibition for detection of nerve agents was developed by G. Guilbaut in 1962 [41] and from this one a lot of ChE electrochemical biosensors were developed for organophosphorus and carbamate insecticides [42,43,44].

By determining the differences in enzyme activity with or without the presence of an inhibitor form the basis of analyte detection, according to the Equation (2):

I% = [ (A_0_ − A_i_)/A_0_] × 100 (2)

where A_0_ is the activity without an inhibitor, and A_i_ is with an inhibitor.

While many studies have looked at the effect of single pesticides on AChE, the effect of mixtures of pesticides still requires extensive investigation. This is important to evaluate the cumulative risk in the case of simultaneous exposure to multiple pesticides. Mwila *et al.* studied the effect of five different pesticides (carbaryl, carbofuran, parathion, demeton-S-methyl, and aldicarb) on AChE activity to determine whether combinations had an additive, synergistic, or antagonistic inhibitory effect. The data from the assays of the mixtures were used to develop and train an artificial neural network. The obtained results indicated that the mixtures had an additive inhibitory effect on AChE activity [45].

Using piezoelectric, different strategies based on inhibition of AChE have been used to increase the sensitivity of biosensor. QCM-based enzyme sensor can be amplified significantly by increasing the mass deposition via the precipitation of enzymatic reaction products.

Abad *et al.* described a biosensor for organophosphorus as well as carbamate pesticides based on the measurement of inhibitory effects of these compounds on the activity of acetylcholinesterase. AChE, immobilized on one of the faces of the crystal and exposing the same to a solution containing a substrate (3-indoyl acetate) converted the substrate to an insoluble product. The rate and extent of the enzymatic reaction can be followed by measuring the frequency changes associated with the mass changes at the QCM surface induced by the enzymatic reaction product. Given that organophosphate as well as carbamate pesticides inhibit AChE activity, the determination of acetylcholinesterase inhibitors such as paroxon or carbaryl can be carried out by following the diminution of the signal from levels found in their absence. Detection limits of 5.0 × 10^−8^ and 1.0 × 10^−7^ M were obtained for paroxon and carbaryl, respectively [16].

Alfonta *et al.* described a three-enzyme layered assembly consisting of horseradish peroxidase, choline oxidase, and acetylcholine esterase. AChE is used to sense acetylcholine by the HRP-mediated oxidation of 3,3*'*,5,5*'*-tetramethylbenzidine, by H_2_O_2_, and the formation of the insoluble product on the transducer. The amounts of generated H_2_O_2_ and the resulting insoluble product on the transducers correlate with the concentration of acetylcholine in the samples. The formation of the insoluble product on electrode supports is followed by faradaic impedance spectroscopy and by cyclic voltammetry [46,47].

Karousos *et al.* reported a similar procedure. Specifically, the authors used a two-enzyme system (acetylcholine-esterase and choline oxidase) which converts acetylcholine to betaine producing hydrogen peroxide as a by-product. In a third enzyme reaction (peroxidase), the peroxide is able to oxidize benzidines into an insoluble product that precipitates out and can adsorb to surfaces. Pesticides inhibit esterase activity thereby reduce the amount of QCM-detectable precipitate produced. The QCM-enzyme sensor system was used to determine carbaryl and dichlorvos down to 1 ppm [17].

Kim *et al.* compared the precipitation of an AChE reaction product from 3-indolyl acetate, over the surface of a QCM-precipitation sensor with respect to enzyme immobilization methods. Under the optimized AChE immobilization, the concentration-dependent decreases in sensor response were measured in the presence of two important and frequently used organophosphate ethyl p-nitrophenyl thionobenzenephosphonate and carbamate carbofuran to evaluate sensitivity of the established sensor system [18].

To avoid complications with the widely studied enzyme-inhibition based sensors resulting from the irreversible nature of the ChE–OP interaction, a novel approach with cholinesterase was proposed from Makower *et al.* The enzyme activity of ChE was not addressed, but the binding of inhibitors to the active site of the enzyme was coupled to the transducer. The binding of tetrameric human butyrylcholinesterase (BChE) with a high molecular weight of 360 kDa to the inhibitor immobilized as a ligand on a piezoelectric quartz crystal was adopted in order to obtain a real-time monitoring of interactions between organophosphate and BChE. For the subsequent detection of organophosphorous pesticide, a competitive affinity assay was developed. Regeneration of the sensing surface was achieved by destroying the chelate complex with EDTA, thus washing out the BChE-inhibitor complex and depositing a new sensing layer. The limit of detection for diisopropylfluorophosphate was 10 nmol/L [19].

Later, Scheller’s research group presented a highly sensitive and rapid sensor that measured the binding of acetylcholinesterase to the reversible inhibitor benzoylecgonine-1, 8-diamino-3, 4-dioxaoctane (BZE-DADOO), a derivative of cocaine. The rate of hydrolysis of cocaine by AChE was almost negligible compared with the diffusion-controlled splitting of acetylcholine. Therefore, the enzyme bound cocaine was split only very slowly and acted as a competitive inhibitor of the hydrolysis of the “natural” substrates. The sensor was successfully tested for detection of diisopropylfluorophosphate inhibitor in river water [20].

Halamek, *et al*. studied the binding of acetylcholinesterase to a propidium-modified piezoelectric quartz crystal and its surface enzymatic activity. Propidium binds to a site remote to the active center of AChE—the peripheral anionic site—which is located on the rim of the gorge to the active site. AChE binding was monitored by a quartz crystal nanobalance, followed by amperometric activity evaluation of the AChE loaded on the sensor. Interestingly, the binding was strong but did not inhibit AChE. However, an excess of propidium in solution inhibited the immobilized enzyme. The surface enzymatic activities observed depend on the amount of enzyme and differ according to the type and species [48].

A macromolecular polymer and carboxyl multi-wall carbon nanotubes (MWNTs-COOH) coated with acetylcholinesterases on the Ag-coated crystal surfaces was presented from Shang *et al.* The authors determined pesticide residue in freshly picked radishes 4 and 8 days post application of phoxim and chlorpyrifos. The sensitivity of the method was compared with gas chromatography. The results show that there was no significant difference between the two methods [49].

Inhibition-based methods have unfortunately some disadvantages and can give to false positives as handling and storage could cause loss of enzyme activity. As a result, baseline testing prior to sample application must be carried out which lengthens testing time. Furthermore, many pesticides irreversibly inhibit an enzyme such as AChE and, therefore, regeneration of the sensor is required after each sample, which can further extend testing time.

### 5.2. Detection of Pesticides Using Immunosensor

Immunosensors are based on the binding interactions between immobilized biomolecules (Ab or Ag) on the transducer surface with the analyte of interest (Ag or Ab), resulting in a detectable signal. The sensor system takes advantage of the high selectivity provided by the molecular recognition characteristics of an Ab, which binds reversibly with a specific Ag. In solution phase, Ab molecules interact specifically and reversibly with an Ag to form an immune complex (Ab − Ag) according to the following equilibrium Equation (3):

Ab + Ag ⇔ Ab − Ag
(3)

where [Ab] represents the antibody concentration, [Ag] the antigen concentration and [Ab − Ag] the antigen-antibody complex concentration.

When the antibody is added in a solution containing the antigen, binding sites of antibody are involved in the antigen interaction; this reaction is regulated by antigen concentration in the sample and by the equilibrium constants. This assumption can be used when antibody and antigen are homogeneous, when the antigen has only one epitope and the antibody has only one binding site and the separation between the bound and the free forms is complete. Immunosensors, which usually incorporate antibodies immobilized on solid matrix do not show the same kinetics of reaction established for those reactants in solution. Immobilization of biomolecules on a surface can alter the properties of the reactants or the surface characteristics.

Piezoelectric immunosensors are devices based on materials such as quartz crystals with bioreceptor, such as Ag or Ab, immobilized on their surface, which resonate on application of an external alternating electric field. The resonant frequency has a proportional relation to the mass changes of the quartz crystal. The biospecific reaction between the two interactive molecules, one immobilized on the surface and the other free in solution or, can be followed in real time.

The first piezoelectric immunosensor was developed by Shons *et al.* [50]. The crystal was precoated with nyebar C and BSA and was used to detect BSA antibodies. The main drawbacks associated with these sensors are a lack of reproducibility in antibody immobilization on the crystal surface and the viscous drag experience in the liquid phase. In order to overcome the latter problem, the resonance frequency of the crystal can be determined in air prior to the addition of sample. The crystal may then be dried, and any change in resonant frequency due to biospecific binding can be calculated. This is known as the ‘dip and dry’ method [51,52].

Hamacek *et al*. reported a similar procedure based on the formation of affinity complexes between an immobilized cocaine derivative and an anti-cocaine antibody or cholinesterase. For both binding reactions benzoylecgonine-1,8-diamino-3,4-dioxaoctane was immobilized on the surface of the sensor. The detection of cocaine was based oil a competitive assay [53].

Jia *et al.* reported a cost-effective protocol to fabricate acoustic micro-immunosensors to measure simultaneously carbofuran and atrazine. Specifically, the patterned quartz crystal is prepared via an electron beam evaporation of gold through a commercial transmission electron microscope grid as a mask. Two different patterned zones on the crystal electrode surface were functionalized either with the monoclonal anti-carbofuran or with anti-atrazine IgG antibody by using thiol chemistry. Different concentrations of antigens were deposited onto their corresponding antibody modified zones monitoring *in situ* the specific interaction between antibody and its antigen from both resonant frequency and resistance changes *versus* time. It was found that the proposed methodology allows sequential specific detection of 4.5 μM and 4.6 μM atrazine, respectively [21].

Pan, *et al.* described the synthesis and characterization of a multi-wall carbon nanotube/poly(amidoamine) dendrimer (MWCNT-PAMAM) hybrid material and its application in the fabrication of piezoelectric immunosensing platform. The aim of modifying PAMAM dendrimers onto the MWCNT surface was to improve its antibody adsorption and dispersion in aqueous phase, which was contributed to enhance the sensitivity and stability in piezoelectric immunoassay procedure. The immunosensor was used for the determination of amounts of a carbamate pesticide-metolcarb in spiked apple and orange juice samples analysis [22].

Funari *et al.* used a photonic immobilization technique to develop an immunosensor for the detection of parathion. An antibody solution was activated by breaking the disulfide bridge in the triad Trp/Cys-Cys through absorption of ultrashort UV laser pulses. The free thiol groups so produced interact with gold lamina making the antibody oriented upside, that is, with its variable parts exposed to the environment, thereby greatly increasing the detection efficiency [23].

A piezoimmunosensor was developed for the determination of the insecticide carbaryl and 3,5,6-trichloro-2-pyridinol (TCP), the main metabolite of the insecticide chlorpyrifos and of the herbicide triclopyr. The detection was based on a competitive conjugate-immobilized immunoassay format using monoclonal antibodies. The good sensitivity, specificity, and reusability achieved, together with the short response time, allowed the application of this immunosensor to the determination of carbaryl and TCP in fruits and vegetables at European regulatory levels, with high precision and accuracy [24].

The significant improvement of QCM is the use of electro-acoustic biosensor based on a film bulk acoustic resonator. A shear mode ZnO film bulk acoustic resonator with a micro-machining structure as a mass-sensitive transducer for the real-time detection of carbaryl was developed from Zhang *et al.* In order to obtain an ultra-low detection level, the artificial antigens were immobilized on the sensing surface of the resonator to employ a competitive format for the immunoassays. The competitive immunoreactions can be observed clearly through monitoring the frequency changes. The presence of pesticide was detected through the diminution of the frequency shift compared with the level without pesticides. The limit of detection for carbaryl is 2 × 10^−10^ M [25].

### 5.3. Detection of Pesticides Using Molecular Imprinting Polymers

New synthetic receptors as molecular imprinting polymers (MIP) are used for immunosensing design. In particular, the molecular imprinting technology is derived from the concept of creating designed recognition sites in macromolecular matrices by means of template polymerization. This technique is based on the *in situ* co-polymerization of cross-linkers and functional monomers that form complexes with template (imprinted) molecules prior to polymerization. After removing the template molecules from the polymerized material, binding sites are left behind, showing complementarity to the template in a subsequent rebinding experiment [26,54].

A piezosensor using molecularly imprinted polymer was developed for fast and onsite determination of pirimicarb in contaminated vegetables. Three MIPs particles were prepared by conventional bulk polymerization and precipitation polymerization in chloroform. The sensor fabricated with MIP-particles exhibited good selectivity and high sensitivity to pirimicarb [27].

Later, Liu *et al*. described a highly selective and sensitive quartz crystal microbalance realized by mixing with polyvinyl chloride and molecularly imprinted polymer microspheres for rapid endosulfan detection. Detection of pesticide in water and milk samples was observed with recoveries of 96.0–104.1% and 101.8–108.0% respectively [28].

Ozkutuk *et al.* presented a sensor based on the modification of molecular imprinted film for the determination of paraoxon. The study also included the measurement of binding interaction quartz crystal microbalance sensor, selectivity experiments and analytical performance of quartz crystal microbalance [29].

## 6. Conclusions

This article reviews the technical status of piezoelectric biosensors and addresses applications of different methodologies for the detection of pesticides in real samples.

Enzymatic methods have been explored as a method of detection of pesticides. This review has attempted to summarize the literature on the use of different enzymes for detection of pesticides. The most published research has focused on the use of AChE for detection of specifically organophosphate and carbamate pesticides.

The reports cited in this overview show the advantages of these systems. However, their sensitivity remains lower than that achieved by other biosensor devices. To meet the requirements of the strict European regulations for the presence of pesticide residues in environment, further studies to improve the QCM sensor sensitivity are necessary.

Future works could be focused on develop new detection systems under the areas of micro-/nanoelectronics; improving the integration and automation level and multianalyte detection. In addition, new synthetic receptors such as aptamers and affibodies will provide complementary excellent options to antibody-based assays.

All these trends are important and should occur in parallel for biosensors development. With the great research efforts, we can expect that in the future smaller and more sophisticated portable and low-cost biosensors.

In many cases, piezoelectric biosensors can be designed for detecting pesticides without the need of expensive or hazardous labels. They offer advantages such as real-time output, high sensitivity, simplicity of use, and cost-effectiveness. The main problem associated with these immunosensors is a change from a sample with low ionic strength to a sample with high ionic strength and vice versa. Moreover, there is a long time needed to reestablish a stable baseline due to changes in viscosity and other parameters close to the QCM surface. Finally, appropriate field-evaluation studies are needed to ascertain the performance of the sensor platforms under a broad range of robust conditions for the analysis of real samples.
